# Zoonotic Epidemic of Sporotrichosis: Cat to Human Transmission

**DOI:** 10.1371/journal.ppat.1006077

**Published:** 2017-01-19

**Authors:** Isabella Dib Ferreira Gremião, Luisa Helena Monteiro Miranda, Erica Guerino Reis, Anderson Messias Rodrigues, Sandro Antonio Pereira

**Affiliations:** 1 Laboratory of Clinical Research on Dermatozoonosis in Domestic Animals, Evandro Chagas National Institute of Infectious Diseases, Oswaldo Cruz Foundation (Fiocruz), Rio de Janeiro, Rio de Janeiro, Brazil; 2 Cell Biology Division, Department of Microbiology, Immunology and Parasitology, Federal University of São Paulo (UNIFESP), São Paulo, São Paulo, Brazil; McGill University, CANADA

## Introduction

Most of the 51 species embedded in the genus *Sporothrix* are nonpathogenic environmental fungi that are closely related to decaying wood, plants, and soil. However, members of the *Sporothrix schenckii* complex are highly successful mammal pathogens, including *S*. *brasiliensis*, *S*. *schenckii sensu stricto (s*. *str*.*)*, *S*. *globosa*, and *S*. *luriei*, the causative agents of human and animal sporotrichosis [1]. Their key to success during mammal infection lies at least in part with their ability to change from a mycelial saprophytic lifestyle at 25°C in the environment to a parasitic yeast cell at an elevated temperature (35°C–37°C), such as those developed by warm-blooded hosts [2].

Typically, infection develops after traumatic inoculation of contaminated soil, plants, and organic matter into skin or mucosa. Alternatively, infection may occur during the animal transmission (cat–cat or cat–dog) and zoonotic transmission (cat–human), which has been mostly associated with scratches or bites from infected cats [2]. In Brazil, *S*. *brasiliensis* is repeatedly associated with feline infection and has consistently shown higher virulence during epizootics, as well as in murine models of sporotrichosis. A hallmark of *S*. *brasiliensis* infection is its tendency to escalate to outbreaks or epidemics among cats with high potential for zoonotic transmission.

Sporotrichosis is an emergent disease and, over the past two decades, the incidence of zoonotic sporotrichosis has been on the rise, particularly in Brazil. Judging from the epizootic and zoonotic epidemics taking place in Rio de Janeiro, Brazil, tackling sporotrichosis requires the engagement of animal and human health policies to reduce the transmission chain of *Sporothrix*.

## Infected Cat: Key Point in Zoonotic Transmission

**Fig 1:**
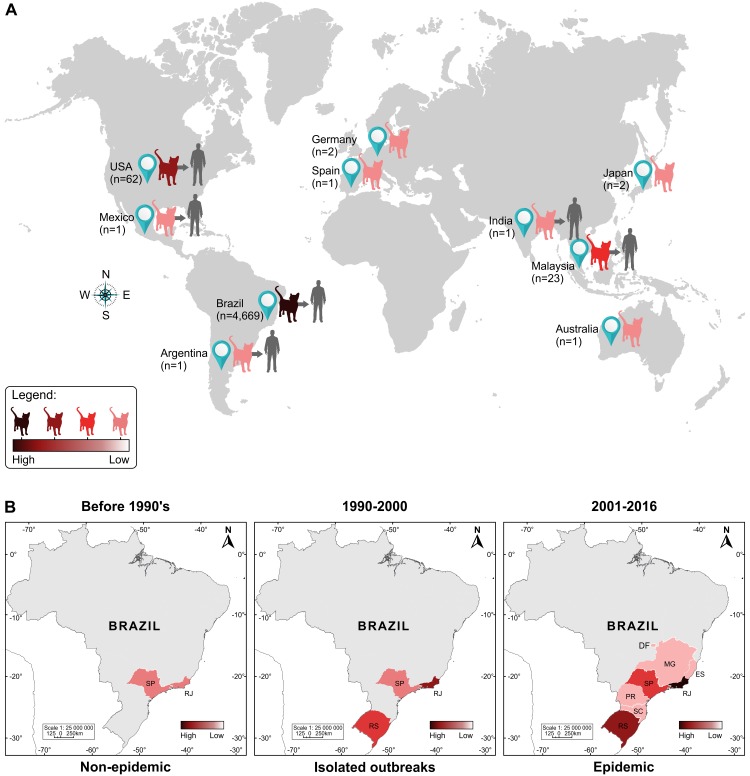
Feline sporotrichosis cases around the world, 1952–2016. (A) Since the mid-20th century, feline sporotrichosis has typically occurred in isolated cases and small outbreaks, and only a few reports of zoonotic transmission have been described in the literature. The Southeast Brazil region has the largest absolute number of cases with an overwhelming prevalence of *S*. *brasiliensis* during epizootic outbreaks. Outside Brazil, most feline cases are due to the classical agent *S*. *schenckii*. (B) Spatiotemporal evolution of feline sporotrichosis cases in Brazil. Over the last two decades (1998–2016), Brazil has experienced a long-lasting outbreak of cat-transmitted sporotrichosis in Rio de Janeiro, with 4,669 cases reported. Cat-borne sporotrichosis due to *S*. *brasiliensis* often appears in the form of outbreaks or epidemics within a short period of time. Remarkably, before the 1990s, Rio de Janeiro reported a low number of cases, nearly always unrelated to feline transmission types.

Cat-transmitted sporotrichosis has been documented in isolated cases or in small outbreaks in the American and Asian continents. Interestingly, although isolated cases of feline sporotrichosis have been documented in Australia, Spain, Japan, and Germany, there are no reports of zoonotic transmission from these regions [3–6] (Fig 1A).

In the United States, isolated cases or small outbreaks were reported from 1952 to 2011, and *S*. *schenckii* was the etiologic agent [7–9]. In Mexico, where the predominant etiologic agent is *S*. *schenckii s*. *str*. [10], only one case of zoonotic sporotrichosis was described related to a scratch of an infected cat in 2008 [11].

Between 2011 and 2014, four cases of human sporotrichosis related to cats were reported in Buenos Aires, Argentina. To our knowledge, this is the first report of zoonotic sporotrichosis in this country [12]. Even though the causative species has not yet been reported, the proximity to the Southern region of Brazil and the type of transmission suggests that *S*. *brasiliensis* may be the species involved in these cases.

In Malaysia, 12 cases of zoonotic transmission related to cats were reported between 1990 and 2010, and five of them were from a small outbreak [13–14]. Recently, 18 clinical isolates from cats in Malaysia were identified as *S*. *schenckii s*. *str*., the prevailing causative agent of the feline sporotrichosis in this country [10]. In India, one case of zoonotic transmission was reported in 2009, and samples from the patient and his cat were positive for *S*. *schenckii s*. *str*. [15].

Although cats are a source of infection and a key to transmission, the evaluation of the feline population in endemic areas were performed in Brazil and Peru [16–17]. Curiously, in an endemic region in Peru, where zoonotic transmission was not recorded, *Sporothrix* was isolated from the nasal cavity and/or nails of two cats (2.38%) without clinical signs of sporotrichosis [17].

## An Unprecedent Zoonotic Epidemic

From 1997 to 2011, 4,188 human cases were recorded at Oswaldo Cruz Foundation (Fiocruz), Rio de Janeiro, the main referral center for the treatment of this mycosis in Brazil [18]. Since 1998, 244 dogs were diagnosed through 2014 [19], and 4,703 cats were diagnosed through 2015. Due to the high incidence of feline sporotrichosis, Rio de Janeiro is presently considered hyperendemic for cat-associated sporotrichosis [2]. However, these cases were recorded from only one institution, so they do not truly reflect the actual picture of this disease in this region. Cases of feline sporotrichosis and zoonotic transmission have been reported in other Brazilian states, especially in Rio Grande do Sul and São Paulo; however, the reported number in these regions is much smaller compared to Rio de Janeiro [2] (Fig 1B). Despite the fact that this is the largest number of canine sporotrichosis cases ever documented, there were no reports of zoonotic transmission from dogs in the Rio de Janeiro epidemic. Dogs are not directly involved in the transmission of the *Sporothrix* spp., probably due to the scarcity of fungal organisms in their lesions in the majority of the cases [20].

## Overcoming Drug Resistance: A Treatment Challenge

Treatment usually requires long-term administration of itraconazole, potassium iodide, or amphotericin B, depending on the severity and location of the lesions. Genotyping of *Sporothrix* during cat-transmitted sporotrichosis associated with antifungal susceptibility profiles raised concern for the emergence and spread of drug-insensitive strains [21–22]. Remarkably, studies reported an increasing number of amphotericin B and itraconazole-insensitive strains over time [21]. Identifying these epidemiological trends associated with the emergence of drug resistance is important to adjust antifungal therapy and to encourage the development of new drugs to treat sporotrichosis. Currently, potential alternative therapies to impair *Sporothrix* development and tackle sporotrichosis include terpinen-4-ol and farnesol [23], miltefosine [24], TCAN26 (a structural analogue of miltefosine) [25], and H3 (a 24-sterol methyltransferase inhibitor) [26].

Successful treatment outcomes will also rely on rapid and accurate diagnosis, especially when dissimilar antifungal susceptibility profiles are noted among different *Sporothrix* species. A cost-effective option includes the molecular diagnosis based on species-specific PCR using primers targeting the calmodulin-encoding gene from pathogenic *Sporothrix*. This PCR-based method is able to detect *Sporothrix* DNA with high sensitivity and specificity from infected specimens even in the presence of DNA from the warm-blooded host [27].

## A Highly Virulent Pathogen Meets a Susceptible Host

Cell-mediated immunity is thought to play an important role in the control of feline sporotrichosis, since increased percentages of CD4 cells are correlated with single lesions, well-organized inflammation, and lower fungal burden. However, most cats with sporotrichosis display lesions with poorly-formed granulomas and high fungal burden, which generally correlated with CD8^low^ cell subsets [28]. Interestingly, this subset is referred to as greatly enhanced by a Th2-shifted environment [29], while the host protective immunity to fungal infections seems to rely largely on a Th1-biased response [30]. Still, the underlying mechanisms leading to different presentations of feline sporotrichosis are undetermined.

Severe feline sporotrichosis may develop independently of retrovirus coinfections, which are known to cause immunosuppression in cats. In most of the endemic areas of Rio de Janeiro, the cats are usually allowed to roam outdoors, and most of them are not vaccinated nor neutered and do not receive regular prophylactic deworming. Therefore, the population of stray cats is large, and the contribution of other infectious diseases to the susceptibility of cats to sporotrichosis cannot be ruled out. In fact, fungal and parasitic infections elicit conflicting immune pathways that may counterbalance each other. In contrast to the antifungal immunity, the classical defense mechanisms to helminth infections include increase of IL-10 and Th2 cytokines, with the suppression of Th1 cytokines [31]. Therefore, we expect this kind of parasite–*Sporothrix* coinfection to increase susceptibility to sporotrichosis. Accordingly, changes in cytokine profile and the lack of fungal clearance are reported in rats with experimental sporotrichosis coinfected by *Taenia taeniaeformis* [32]. We have no clear information about which cytokines drive mechanisms of immune response in cats with sporotrichosis and whether helminth coinfection shifts the inflammatory environment. Even so, we hypothesize that helminth infection control in cats with sporotrichosis could balance the immune response in favor of a Th1 type and contribute to the fungal clearance with a reduction on their zoonotic potential.

It is also possible that an increased virulence of *S*. *brasiliensis* may be a factor contributing to the sporotrichosis outbreak in Rio de Janeiro. Experimental model systems from mice to invertebrates have been used to investigate the emergence of pathogenicity of *Sporothrix* and bring attention to differences in pathology and virulence factors, where *S*. *brasiliensis* usually display higher fungal burden, invasiveness, and extensive tissue damage when compared to the remaining agents in the *S*. *schenckii* complex [33]. In support of this hypothesis, an increasing virulence of *S*. *brasiliensis* over the years of infection was reported in a patient and assigned to improve fungal resistance to host’s oxidative stress [34].

Recently, the distinct sensititivities to oxidative stress between *S*. *brasiliensis* and *S*. *schenckii* were correlated with the Hog-1 stress-induced kinase pathway [35]. The production of melanin is also associated with resistance to phagocytosis in more pathogenic isolates of *Sporothrix*, notably to *S*. *brasiliensis* [36]. Cell wall lipids are also able to inhibit the phagocytosis [37], and yeasts of *Sporothrix* are more virulent than longer-term culture conidia, which is thought to reflect enhanced evasion of phagocytosis [38]. Unlike classic transmission of *Sporothrix*, due to inoculation of asexual spores, zoonotic transmission is thought to be mediated by yeast cells. Innate immunity plays a role in the development of adaptive response, by recognizing fungal wall elements through Toll-like receptors [37]. Thus, the direct inoculation of yeast could lead to more challenging host–pathogen interactions and be crucial for the establishment of the infection. We speculate that the evasion of the yeast cells could take part in the typical development of lesions [28] with macrophages apparently unable to complete phagocytosis in feline sporotrichosis and prevent further activation of adaptive immunity. Fungal wall compounds are also important in the adhesion to extracellular matrix, in particular 3-carboxymuconate cyclase (glycoprotein Gp70 and Gp60) [39–40]. The glycoprotein Gp60 has also been suggested to act as a virulence factor, as it is associated with more virulent isolates of *Sporothrix*, principally *S*. *brasiliensis* [40].

## Vaccine Candidates and Perspectives

A specific and protective humoral response against *Sporothrix* has already been observed [40–41] and may be a clue for the further investigation of vaccine candidates. *Sporothrix* 3-carboxymuconate cyclase has emerged as a potential target for vaccination studies [40,42]. Since both Gp70 and Gp60 are recognized by antibodies in serum from cats with sporotrichosis [40], and considering that these cats are a potent and major source of *Sporothrix* in Brazilian epidemics, an effective vaccine to prevent their infection or control their fungal burden would be a principal measure to reduce transmission.

Studies to identify a vaccine candidate and the ideal adjuvant are ongoing [40,43]. Promising results using *S*. *schenckii* cell wall protein in an aluminum hydroxide (AH) adjuvanted formulation have been reported [43]. However, the use of AH as an adjuvant should be carefully reviewed for future studies in cats, since this compound is involved in feline vaccine-induced sarcomas [44]. Passive immunization or the nonadjuvanted formulation should therefore be studied initially. Even though, the recent progress in the search for vaccine antigens and adjuvants herein discussed is encouraging especially as an important aid for controlling the enormous health burden of feline sporotrichosis in endemic areas [42]. Therefore, we raise the cats as the key host for further vaccine investigations.
